# Nutritional value, antioxidant and antidiabetic properties of nettles (*Laportea alatipes* and *Obetia tenax*)

**DOI:** 10.1038/s41598-020-67055-w

**Published:** 2020-06-17

**Authors:** Nomfundo Thobeka Mahlangeni, Roshila Moodley, Sreekantha Babu Jonnalagadda

**Affiliations:** School of Chemistry and Physics, University of KwaZulu-Natal, Durban, South Africa

**Keywords:** Secondary metabolism, Analytical chemistry

## Abstract

Nettles are commonly consumed in South Africa, Europe and Asia and are used in traditional medicine to treat a variety of ailments. In this study, the nutritional value of the leaves of nettles (*Laportea alatipes* and *Obetia tenax*) was evaluated and compared, when cooked and uncooked. The results showed a decrease in the concentrations of crude protein, vitamin A, vitamin E and metals after cooking of nettles. Although cooking reduced the concentrations of essential elements in nettles, their contribution to the diet remained adequate. *L. alatipes* presented with reduced levels of Cd (from 1.86 to 0.810 mg kg^−1^) and Pb (from 2.87 to 1.88 mg kg^−1^) after cooking. Similarly, Cd (from 2.97 to 0.780 mg kg^−1^) and Pb (from 2.21 to 0.795 mg kg^−1^) levels in *O. tenax* decreased after cooking, demonstrating the significance of cooking. The antioxidant activity of the nettles was determined using the 2,2-diphenyl-l-picrylhydrazyl (DPPH) free radical and ferric reducing antioxidant power (FRAP) assays. The methanol extract of *Obetia tenax* showed high ferric reducing power whilst the radical scavenging activity was due to the presence of the bioactive molecule, β-carotene, in the plants which exhibited higher DPPH radical scavenging ability relative to test samples and standards. The *in vitro* antidiabetic activity of the extracts and compounds from the nettles was better than or comparable to that of the known standard, acarbose, which underscores the prospective antidiabetic properties of nettles. Overall, our study provides scientific validation for the ethno-medicinal use of nettles and supports their consumption, which highlights their potential as nutraceuticals.

## Introduction

Malnutrition results from inadequate intake of food or an improper diet and it affects normal functioning of the human body as well as growth and development in children^1^. Malnutrition is a serious health concern in developing countries such as Asia, Africa, Latin America and the Middle East with one in five people being malnourished and experiencing the conditions associated with micronutrient deficiencies^2^. For proper nourishment, the human body needs foods rich in macronutrients (carbohydrates, fats, proteins and vitamins) and minerals but low in sugar. In South Africa, non-communicable diseases (NCDs) such as cardiovascular disease, chronic respiratory disease and cancer account for 37% of deaths. The World Health Organization (WHO) has therefore suggested a daily intake of 400 g of fruits and vegetable per day to reduce the risk of NCDs^3,4^.

Food insecurity also plays a role in malnutrition as nutritious foods are inaccessible, unaffordable or unavailable. Leafy green vegetables are important in developing countries because they are cheap, readily available, nutritious and easy to cook. These leafy green vegetables contain substantial amounts of antioxidant vitamins (β-carotene, vitamin C and E) and minerals^5^.

Diabetes mellitus is a metabolic disorder characterized by hyperglycemia (high blood glucose) resulting in defects in the secretion of insulin, impaired action of insulin or both^6^. In the absence of insulin, glucose (from broken down carbohydrates and starch) builds up in blood vessels as it cannot be absorbed into the cells of the body which results in organ and tissue failure. The International Federation of Diabetes (IDF) reported that about 15.9 million have diabetes in Africa, and expected to increase by more than 100% in 2045^7^. South Africa is amongst the top five countries with the highest number of people living with diabetes^7^.

The growing popularity of nutraceuticals has led to a greater demand for the identification of new plants that are both nutritional and medicinal. *Laportea alatipes* Hook. f. and *Obetia tenax* (N.E.Br.) Friis are from the Urticaceae (nettle) family and are known for their nutritional and medicinal value. These nettles are found in KwaZulu-Natal, South Africa. They are generally known as forest nettle (*L. alatipes*) and mountain nettle (*O. tenax*) or Imbati in isiZulu. In traditional medicine, nettles are used to treat conditions such as rheumatoid arthritis, gout, eczema, benign prostatic hyperplasia, anemia, influenza, asthma and diabetes^8,9^.

Previously, we reported on the distribution of nutrients and anti-nutrients in the nettles, *L. peduncularis* susp. *peduncularis* and *Urtica dioica*^10^. In this study, we investigate the concentrations of nutrients and anti-nutrients in the nettles, *L. alatipes* and *O. tenax* and compare these values to those obtained from our previously study. The impact of cooking on nutritional value is also evaluated. Antioxidant and antidiabetic properties of the nettle extracts and isolated compounds were also investigated.

## Materials and Methods

The materials and methods section is presented as Supplementary Material 1.

### Human and animal studies

This article does not contain any studies with human or animal subjects.

## Results and Discussion

### Elemental analysis

On average, the moisture content of *L. alatipes* was found to be 79.2% and that of *O. tenax* was found to be 82.1%. Food processing such as cooking alters the nutritional value of uncooked plant foods. For this reason, both uncooked and cooked leaves of *L. alatipes* and *O. tenax* was investigated for essential and toxic metal levels to determine their nutritional value and to assess for metal toxicities, respectively (Table 1).

**Table 1:** Concentration (mg kg^−1^, mean ± SD, n = 4) of essential and toxic elements in *L. alatipes* and *O. tenax* leaves (uncooked (UC) and cooked (C)).

	Laportea alatipes		Obetia tenax		DRI		*L. alatipes*	*O. tenax*	*L. peduncularis*	*U. dioica*
	UC	C	UC	C	RDA^b^ (mg per day)	DV^c^ (mg)	UC/C	UC/C	UC/C	UC/C
(mg kg^−1^)							(mg per 60 g)			
**Macro-elements**										
Ca	34084 ± 1074ca	12652 ± 1598a	21999 ± 1618b	21620 ± 636b	1000–1300	1000	2045/759	1320/1297	1654/770	1899/871
Fe	57115 ± 6413c	39196 ± 5376a	112534 ± 9902d	16807 ± 2035b	8–18	18	3427/2352	6752/1008	78.7/60.6	12.5/19.1
Mg	12407 ± 432c	2023 ± 212a	10648 ± 544d	4075 ± 155b	240–400	400	744/121	639/245	420/142	371/136
P	1715 ± 108c	1264 ± 112a	1340 ± 74.6d	1135 ± 153b		1000				
**Micro-elements**										
Ba	77.0 ± 5.22b	57.7 ± 6.26a	151 ± 7.41c	88.1 ± 5.00b						
Co	1.74 ± 0.26a	1.50 ± 0.19a	6.64 ± 0.430c	0.845 ± 0.118b						
Cr	12.1 ± 1.32b	0.073 ± 0a	87.7 ± 6.11c	0.074 ± 0.001a	0.02–0.035	0.120	0.726/0.004	5.26/0.004	0.186/0.407	0.063/0.1
Cu	19.1 ± 1.02b	12.8 ± 0.59a	23.9 ± 2.01c	14.0 ± 0.86a	0.7–0.9	2	1.15/0.768	1.43/0.840	1.38/0.382	1.05/0.97
Mn	260 ± 18.2c	180 ± 24.9a	206 ± 14.3a	76.1 ± 3.34b	1.6–2.3	2	15.6/10.8	12.4/4.57	91.3/10.5	1.53/2.93
Ni	11.8 ± 0.95b	6.36 ± 1.39a	15.8 ± 1.04c	8.42 ± 1.19a	ND		0.708/0.382	0.948/0.505	0.287/0.121	0.144/0.03
Zn	60.7 ± 4.51c	50.9 ± 4.31a	34.3 ± 0.705b	35.2 ± 2.06b	8–11	15	3.64/3.05	2.06/2.11	2.25/1.51	1.9/1.6
**Toxic elements**										
Cd	1.86 ± 0.207b	0.810 ± 0.168a	2.97 ± 0.177c	0.780 ± 0.167a						
Pb	2.87 ± 0.250c	1.88 ± 0.134a	2.21 ± 0.128a	0.795 ± 0.157b						

^a^Values in the same row with different letters are significantly different (Tukey’s post hoc comparison, *p* < 0.05), ^b^Indicates recommended dietary allowance^14^, ^c^Indicates daily values^20^, ^d^Results of previous study^10^.

Dietary reference intakes (DRIs) (recommended dietary allowance (RDAs) and daily values (DVs)) and average concentration (in mg per 60 g, dry mass, n = 4)) of essential elements in nettles (*L. alatipes*, *O. tenax*, *L. peduncularis*^d^ and *U. dioica*^d^).

Ingestion of toxic metals, even at low concentrations, can be detrimental to human health. Cadmium is known to target the kidneys and respiratory system^11^, and long-term exposure to Pb may cause damage to the nervous system and can lead to blood disorders^12^. Subsequently, the joint Food and Agriculture Organization of the United Nations (FAO) and WHO have set the maximum levels of Cd and Pb in leafy vegetables at 0.2 mg kg^−1^ and 0.3 mg kg^−1^, respectively^13^. In *L. alatipes*, Cd levels reduced by 56% from 1.86 to 0.810 mg kg^−1^ and Pb levels reduced by 34% from 2.87 to 1.88 mg kg^−1^ whilst in *O. tenax*, Cd levels reduced by 73% from 2.97 to 0.780 mg kg^−1^ and Pb levels reduced by 64% from 2.21 to 0.795 mg kg^−1^, after cooking. This study shows concentrations of toxic elements, Cd and Pb, to decrease significantly after cooking which indicates the benefits of cooking leafy vegetables to reduce metal toxicities.

The results in Table 1 also show the concentrations of elements in 60 g (suggested serving size which is equivalent to two cups of spinach) of *L. alatipes*, *O. tenax* (this study), and *L. peduncularis* and *U. dioica* and their contribution to recommended dietary allowances (RDAs) and daily value (DVs) for the studied essential elements^10,14^. The estimated contribution to the diet by nettles can be used to assess for elemental value and deficiencies. Low fruit and vegetable intake by individuals has been a major contributor to micronutrient deficiencies, as a result, WHO has recommended consumption of a minimum of 400 g of fruits and vegetables per day^15,16^. Studies have shown that the average consumption of vegetables is less than 80 g per day in South Africa^16,17^.

For the macro and micro-elemental content, the results showed a decrease in *L. alatipes* and *O. tenax* after cooking (Table 1). The most common elements that are deficient in humans are Fe, Zn and Cu^18^. Disorders associated with Zn deficiencies include epilepsy, diabetes, multiple sclerosis, sickle-cell anemia and mood disorders. Insufficient dietary intake of Cu can lead to impaired Fe metabolism with hypochromic anemia and bone abnormalities. The results show nettles to be rich in Fe which would be beneficial to individuals who are suffering from Fe deficiency anemia^19^. After cooking, *L. alatipes* and *O. tenax*, respectively contribute 85% and 93% towards the RDA for Cu, and 28% and 19% towards the RDA for Zn. These values are much higher than those obtained for *L. peduncularis* (42% Cu and 14% Zn) and comparable to those obtained for *U. dioica* (108% Cu and 14% Zn)^10^. The results also show *L. alatipes* and *O. tenax*, respectively to contribute 38% and 42% towards the DV for Cu, and 20% and 14% towards the DV for Zn, after cooking. This shows the nettles, *L. alatipes* and *O. tenax*, to be richer in essential elements compared to *L. peduncularis* and *U. dioica*.

The results of this study show that, while nettles contribute to the dietary reference intakes (DRIs) for essential elements for most individuals, this contribution is reduced on cooking due to leaching of elements into the cooking water. However, nettles were found to be nutritious, whether cooked or uncooked, with cooking being more beneficial due to reduction of toxic metal levels.

The results in Table 2 show the concentrations of macronutrients in the different nettles and their contribution to the RDA and DV for these nutrients. After cooking, *L. alatipes* and *O. tenax*, respectively contribute more to the RDA of proteins (5% and 6%), vitamin C (29% and 31%) and vitamin E (153% and 140%) compared to *L. peduncularis* and *U. dioica*^10^. A similar trend was observed for DVs. This study shows *L. alatipes* and *O. tenax* to have higher macronutrient content compared to *L. peduncularis* and *U. dioica*, after cooking.

**Table 2:** Dietary reference intakes (DRIs) (recommended dietary allowances (RDAs) and daily values (DVs)) of macronutrients for most individuals and average concentrations of macronutrients (n = 4) in *L. alatipes* (LA), *O. tenax* (OT) *L. peduncularis* (LP) and *U. dioica* (UD) leaves (uncooked/cooked).

	Average concentration in uncooked/cooked leaves (g per 60 g, dry mass)				DRI^a^	
	LA	OT	LP^b^	UD^b^	RDA (g per day)	DV^c^ (g)
Carbohydrates	35.1/35.7	30.2/38.4	12.9/27.7	32.0/36.9	130	300
Proteins	3.22/2.94	3.20/3.11	0.82/1.07	0.89/1.10	34–56	50
Vitamin A^d^	9.9 × 10^−2^/9.4 × 10^−4^	1.1 × 10^−2^/1.0 × 10^−2^	—	—	0.6 × 10^−3^–0.9 × 10^−3^	0.7 × 10^−3^–0.9 × 10^−3^
Vitamin C	0.023/0.022	0.023/0.023	0.011/0.010	0.013/0.010	0.045–0.075	0.06
Vitamin E	0.059/0.023	0.067/0.021	0.011/0.014	0.016/0.015	0.011–0.015	0.020

^a^Indicates dietary reference intakes^14^.

^b^Results of previous study^10^.

^c^Indicates daily values^20^.

^d^Vitamin A as retinol; 1 µg β-carotene = 0.167 µg retinol.

Vitamin A deficiency has been shown to be a major nutrient deficiency in South Africa and has been linked to non-communicable diseases such as xerophthalmia, which is associated with dry eyes and night blindness. Vitamin C, an effective antioxidant, is a cofactor in numerous physiological reactions such as collagen gene expression, peptide hormone activation, and carnitine synthesis. Vitamin E is known for its antioxidant activity especially the inhibition of membrane lipid peroxidation^21^.

Of the three vitamins determined, only vitamin C was unaffected by cooking in all four nettles. A study conducted on the effect of cooking on vitamin C content of some selected vegetables showed highest loss of vitamin C in pepper (64.7%) after 30 minutes and lowest loss of vitamin C in spinach (9.9%) after 5 minutes^22^. Vitamin C, a water-soluble and heat labile substance will easily leach into water and then degrade by heat. Elevated temperatures and long cooking times have been found to cause loss of vitamin C^23^. In this study, there was high retention of vitamin C after 15 minutes of cooking. This could be due to shorter cooking times and low temperatures, which could have been inadequate for release of vitamin C from its intracellular locations.

Green leafy vegetables have higher retention of vitamin A than root vegetables, which may be attributed to its increased extractability following denaturation of proteins and a complete breakdown of the cell wall in plants which occur as a result of cooking^22^. In this study, there was lower retention of vitamin A and E after cooking of *O. tenax* and *L. alatipes*. Vitamin A is found in the photosynthetic pigment-protein complexes of chloroplasts in leafy green vegetables, which inhibit its extractability^24^. Green leafy vegetables have vitamin E occurring mainly as α-tocopherol which is situated inside chloroplasts^25^. Cooking could increase extractability by softening plant walls and disrupting protein complexes. Solubilization of vitamins A and E in cellular lipid emulsions formed during cooking could decrease retention by the leaves and lead to lower concentrations.

### Antioxidant and antidiabetic activities

**Figure 1:**
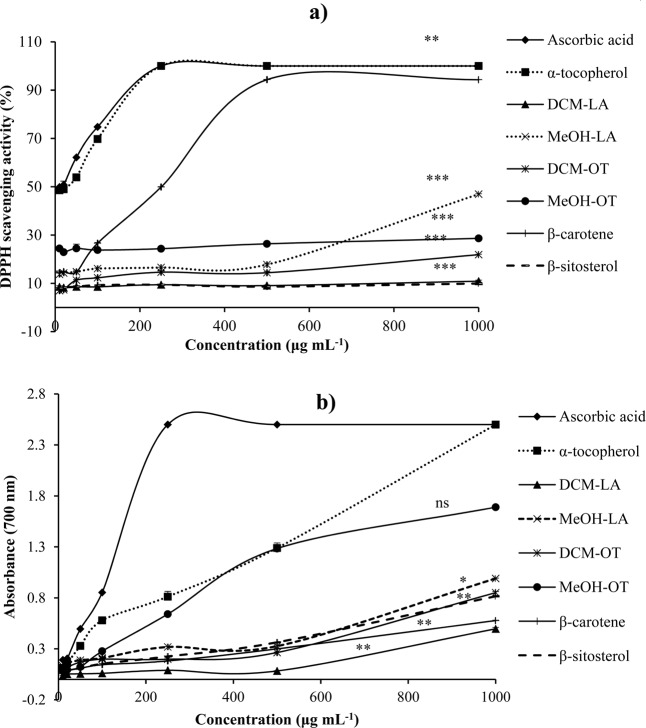
(**a**) DPPH radical scavenging activity and (**b**) Ferric reducing power of *L. alatipes* (LA), *O. tenax* (OT) and isolated compounds. Values represented as mean ± SD, n = 3. One way Anova and Dunnetts post hoc test shows significant difference from positive control, ascorbic acid, a) (**p < 0.01 and ***p < 0.001), b) (ns-not significant, *p < 0.05 and **p < 0.01).

An antioxidant is a biochemical substance that protects living cells from damage caused by free radicals that can cause cancer, cardiovascular diseases and other age-related diseases. The 2,2-diphenyl-l-picrylhydrazyl (DPPH) assay is based on the measurement of the ability of antioxidants to scavenge the DPPH radical in solution as observed by a loss of color from deep violet to yellow^26^. In this study, the methanol (MeOH) and dichloromethane (DCM) extracts, and isolated compounds, β-carotene and β-sitosterol from the nettles, *L. alatipes* and *O. tenax*, was evaluated for antioxidant activity. The extracts showed moderate inhibition of the DPPH radical relative to the known standards, ascorbic acid and α-tocopherol (Fig. 1a). This was also observed in a previous study on the antioxidant activity of the ethanol and hot water extracts of *Laportea interrupta*^27^. The highest antioxidant activity was exhibited by β-carotene (IC_50_ = 339 µg mL^−1^) when compared to the extracts and β-sitosterol, which was comparable to ascorbic acid (IC_50_ = 271 µg mL^−1^).

The ferric reducing antioxidant power (FRAP) assay, which is within the technological reach of most laboratories, evaluates the reduction of Fe^3+^ to Fe^2+^ by the donation of an electron by the antioxidant, and it offers an accepted index of potential antioxidants^28^. In this study, there was a positive relationship between the concentrations of the plant extracts and the compound with absorbance (Fig. 1b). The MeOH extract of *O. tenax* had the highest reducing ability compared to the other extracts however; this was lower than ascorbic acid and α-tocopherol. Moderate antioxidant activity was observed for the other extracts. Unlike the DPPH assay, β-sitosterol showed higher reducing ability than β-carotene.

**Figure 2:**
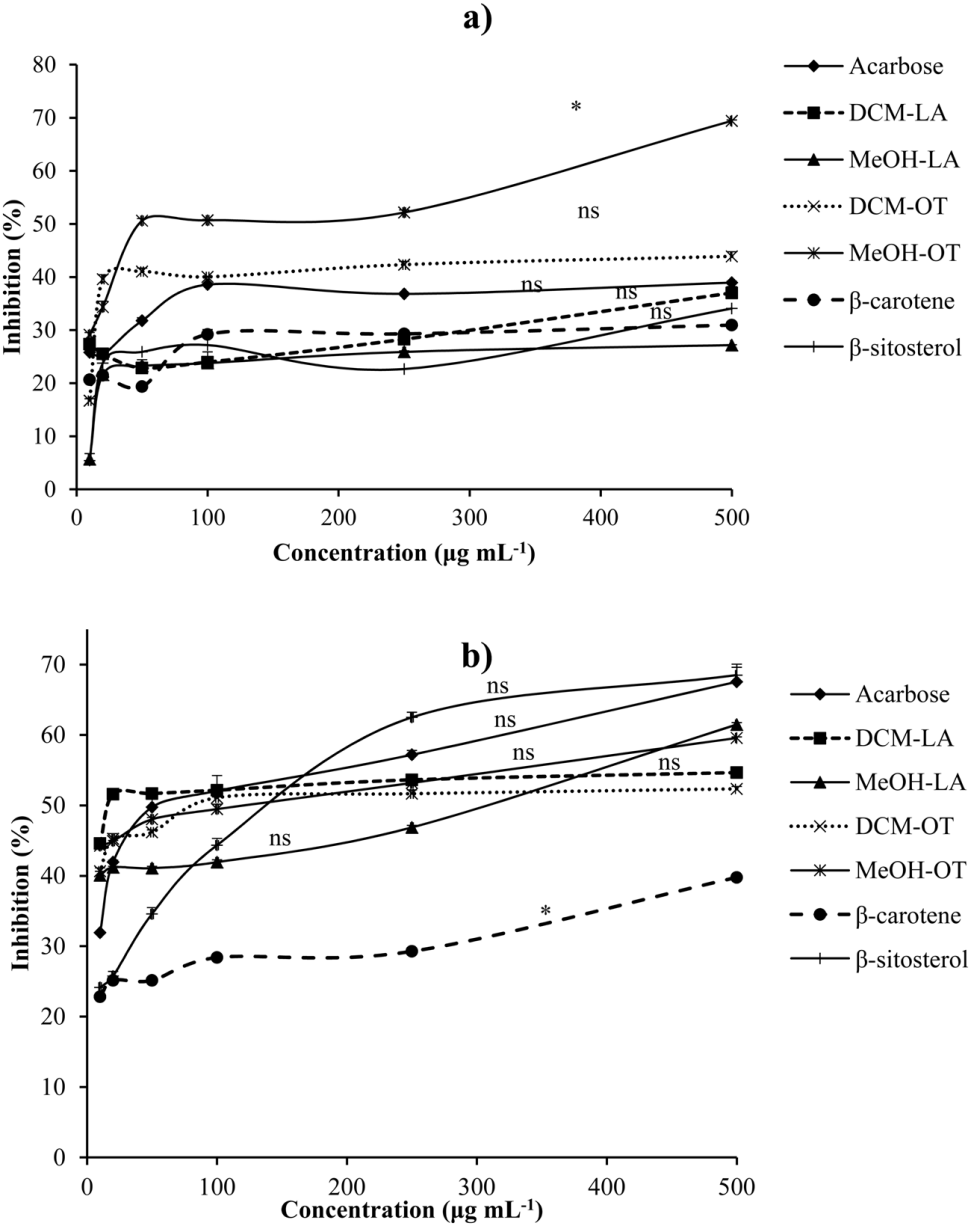
(**a**) Alpha amylase and (**b**) Alpha glucosidase inhibitory activity of *L. alatipes* (LA), *O. tenax* (OT) and isolated compounds. Values represented as mean ± SD, n = 3; One way Anova and Dunnetts post hoc test shows significant difference from positive control, acarbose (ns- not significant and *p < 0.05).

The enzyme responsible for the digestion of dietary carbohydrates in the digestive tract of humans is α-amylase by acting upon the linkage between the glucose units of carbohydrates. α-Amylase hydrolyzes the carbohydrates to disaccharides then to glucose. Inhibition of this enzyme slows down the rate of digestion of carbohydrates, glucose absorption and thereby lowers blood glucose levels, consequently, reducing the postprandial increase of plasma glucose^29^. An inhibitor of α-amylase reduces the conversion of carbohydrates to glucose. The effects of plant extracts and isolated compounds on the inhibition of α-amylase are presented in Fig. 2a. The results showed the response to be dose-dependent. The MeOH extract (IC_50_ = 190 µg mL^−1^) and DCM extract (IC_50_ = 645 µg mL^−1^) of *O. tenax* were shown to be more active than the reference standard, acarbose (IC_50_ = 875 µg mL^−1^). The results suggest that extracts from nettles possess constituents that are able to block the hydrolysis of 1,4 glycosidic linkage of starch into glucose^30^. β-sitosterol (IC_50_ = 1009 µg mL^−1^) had a lower IC_50_ value compared to β-carotene (IC_50_ = 1303 µg mL^−1^). These two compounds were found in *O. tenax* indicating synergistic effects for antidiabetic activity. Extracts of *L. alatipes* showed inhibition of α-amylase but this was lower than acarbose.

The inhibition of α-glucosidase activity of the extracts and compounds from nettles is presented in Fig. 2b. This enzyme is located in the small intestines, catalyzes the digestion and absorption of glucose into the intestines. Inhibition of α-glucosidase decreases the digestion of carbohydrates, thereby decreasing postprandial blood glucose levels. There was moderate inhibition of α-glucosidase compared to α-amylase by *O. tenax*. The inhibitory potential of the DCM extract of *L. alatipes* (IC_50_ = 42 µg mL^−1^) was lower than acarbose (IC_50_ = 154 µg mL^−1^). β-Sitosterol (IC_50_ = 229 µg mL^−1^) was a more active inhibitor of α-glucosidase compared to β-carotene. Previous studies on antidiabetic potential of plants from the Urticaceae family have shown promising results; extracts from *Urtica dioica* were reported to have antidiabetic effects in male rats with fructose-induced insulin resistance and aqueous extracts of *Laportea ovalifolia* were found to have antihyperglycaemic activity on alloxan diabetic rats^31,32^.

## Conclusion

The results showed cooking to reduce the elemental and vitamin concentrations in nettles, which are temperature and time dependent. This reduction in concentration is significant when considering potential metal toxicities due to metals such as Cd and Pb. The radical scavenging activity of the nettles was due to the presence of bioactive molecules in the plant with β-carotene having the highest activity. The FRAP assay indicated synergistic effects as the MeOH extract of *O. tenax* exhibited the highest ferric reducing power. The extracts of the leaves of *O. tenax* had higher α-amylase inhibitory activity whilst those of *L. alatipes* had higher α-glucosidase inhibitory activity. This study underscores the prospective antidiabetic properties of nettles; however, further studies are required to confirm this biological activity. Overall, our study supports the consumption of nettles for nutritional benefit and highlights their potential as nutraceuticals.

## Supplementary information

Supplementary Information.
