# Supplementary material for: Nutritional value, antioxidant and antidiabetic properties of nettles (Laportea alatipes and Obetia tenax)
Source: Sci Rep. 2020 Jun 17;10:9762. doi: 10.1038/s41598-020-67055-w (PMC7300021; doi:10.1038/s41598-020-67055-w)
Supplement: Supplementary file 1 — Supplementary Information. [file 41598_2020_67055_MOESM1_ESM.pdf]

Nutritional value, antioxidant and antidiabetic properties of nettles (*Laportea alatipes* and *Obetia tenax*)

Nomfundo Thobeka Mahlangeni[a], Roshila Moodley[a*], and Sreekantha Babu Jonnalagadda[a]

[a] School of Chemistry and Physics, University of KwaZulu-Natal, South Africa

*Address correspondence to Dr Roshila Moodley, School of Chemistry & Physics, University of KwaZulu-Natal, Westville Campus, Private Bag X54001, Durban 4000, South Africa; Telephone: +27 31 260 8127/3090; Facsimile: + 27 31 260 3091; E-mail: moodleyrosh@ukzn.ac.za

Supplementary Material

1. Materials and Methods

1.1 Plant collection and preparation

Plants (*L. alatipes* and *O. tenax*) were collected from various sites in KwaZulu-Natal, South Africa and were identified by curator, Mr E. Khathi, from the School of Life Sciences, University of KwaZulu-Natal, Westville and voucher specimens (Mahlangeni NT2 and Mahlangeni NT3) were deposited in the ward herbarium at the university. Some of the fresh leaves were subjected to conventional cooking on a hotplate at 70 °C by boiling in distilled water for 15 min then filtering [1]. Thereafter, cooked leaves were dried in an oven at 50 °C. Some of the fresh leaves (uncooked) were dried in an oven at 50 °C. Both cooked and uncooked leaves were crushed using a food blender (Salton, SMS15, Johannesburg, South Africa) and stored in plastic bottles.

1.2 Nutrient analysis

All solvents and reagents were of analytical grade and were purchased from Merck (Darmstadt, Germany) and Sigma Aldrich (St Louis, USA). Nutrient analysis was performed as described in our previous study [1]. The moisture and ash content were established by well-known methods [2,3]. Moisture content was determined by drying fresh sample (approximately 1.00 g weighed in a crucible) in the oven at 105 °C, to constant mass. Thereafter, the crucible with the dried sample was removed from the oven and placed in the desiccator to cool. The percentage moisture was calculated by the following equation:

$$\% \text{ Moisture content} = \left[\frac{W_2 - W_3}{W_3 - W_1}\right] \times 100 \qquad (1)$$

where W_1 is the weight of the crucible, W_2 is the weight of the crucible with the fresh sample and W_3 is the weight of the crucible with the dried sample.

The ash content was determined by igniting the dried sample in a muffle furnace at 600 °C for 12 h. Fat content was determined by the soxhlet method using n-hexane. Briefly, approximately 5 g of dried sample was placed into an extraction thimble and 100 mL of n-hexane was poured into a 200 mL round bottom flask. The soxhlet apparatus was then assembled on a heating mantle set at 60 °C and the extraction process was continued for 6 h. The solvent was removed using a rotary evaporator. The resulting oil was quantified gravimetrically and the percentage oil was calculated using the following equation:

$$\% \text{ Fat content} = \left[\frac{M_2 - M_1}{E}\right] \times 100 \qquad (2)$$

where M_1 is the weight of the empty vessel, M_2 is the weight of the vessel with the fat residue and E is the sample weight.

Protein content was determined by Kjeldahl distillation method, multiplying nitrogen by 6.25 [4]. Total carbohydrate was obtained by difference (subtracting the protein, ash, and fat content from the total dry mass of the sample). The vitamin C content was determined by the iodometric method [5]. Dried samples (approximately 20 g) were placed in a mortar and 20 mL doubly distilled water was added. The mixture was stirred for 20 min and filtered by suction using a Buchner funnel. The filtrate was placed into a 50 mL volumetric flask and made up to the mark with doubly distilled water. A 25 mL aliquot of the leaf solution was placed into a conical flask then 5 mL of 0.15 M potassium iodide, 0.1 M hydrochloric acid and 1 mL of 1% starch solution indicator was added. This was titrated against a potassium iodate solution (0.009 M). The endpoint was observed by the permanent blue-black color due to the starch-iodine complex.

The vitamin A content (β-carotene) was determined using previously described methods [6]. Briefly, approximately 0.5 g of dried sample was mixed with 4 mL cold ethanol and extracted with 8 mL hexane followed by vortex mixing for 2 min then centrifuged at 1000 rpm for 15

min. β-carotene was determined spectrophotometrically (Biochrom Libra S11, Cambridge, England) at 450 nm.

Vitamin E content was determined by previously described methods [7]. About 0.5 g of dried sample was mixed with 4 mL hexane then mixed with 1 mL ethanol and 0.5 mL 25% ascorbic acid and pre-incubated in a water bath at 70 °C for 5 min. Thereafter, 0.5 mL of potassium hydroxide (10 M) was added then further incubated at 70 °C for 30 min. The tubes containing the mixture were then placed in an ice bath and 8 mL of hexane was added and vortex mixed for 1 min. The mixture was then centrifuged at 1000 rpm for 15 min. The organic layer (hexane) was removed and used to estimate the vitamin E content. To the of the organic layer (1.5 mL), 0.2 mL of 0.2% bathophenonanthroline was added and thoroughly mixed while covered with foil. A solution of $FeCl_3$ (0.2 mL, 0.001 M) was added and again mixed for 1 min, after which, 0.2 mL of 0.001 M H_3PO_4 was added and vortex mixed. The absorbance was read at 536 nm using a UV spectrophotometer (Biochrom Libra S11, Cambridge, England). Absolute ethanol served as a blank. A calibration curve of α-tocopherol (prepared in absolute ethanol) at various concentrations was used to determine the vitamin E content in the leaves.

1.3 Phytochemical analysis

Leaves of *L. alatipes* (196 g), and *O. tenax* (292 g) were air-dried, ground and extracted with hexane, dichloromethane (DCM), and methanol (MeOH), in turn, by continuous shaking on an orbital shaker for 48 h. The aqueous MeOH extract was partitioned with DCM followed by ethyl acetate (EtOAc). All mixtures were filtered thereafter the filtrate (extract) was concentrated to dryness and stored in the fridge until analyzed. The extracts were spotted on TLC plates (Merck silica gel 60, 20 x 20 cm F254 aluminum sheets) and were subjected to column chromatography using Merck Kieselgel 60 silica gel (0.063-0.200 mm, 70-230 mesh ASTM).

The hexane and DCM extract of *L. alatipes* leaves were combined due to similar TLC profiles. The combined extract (12.23 g) was loaded onto the column and separated using a hexane: EtOAc step gradient starting from 100% hexane that was stepped by 5% to 100% EtOAc. About 20 mL were collected for each fraction. Fractions with similar retention factors (R_f) on the TLC, were combined and evaporated on a rotary evaporator. Fractions 51-72 eluted with 90% hexane: 10% EtOAc was recrystallized with 100% MeOH to afford compound C1 (12.1 mg) as a white solid.

The DCM extract (5.91 g) of *O. tenax* leaves was separated using a 90% hexane: 10% EtOAc solvent system and afforded compound C1 (12.1 mg) in fractions 4-5. The aqueous MeOH extract (150 mL) of *O. tenax* leaves were partitioned with 150 mL DCM in triplicate then subjected to column chromatography using a DCM: MeOH solvent system. Compound C2 (3.23 mg, orange powder) was eluted with 99% DCM: 1% MeOH.

^{1}H and ^{13}C nuclear magnetic resonance (NMR) spectra were recorded on a Bruker Avance III spectrometer at 400 MHz in deuterated chloroform ($CDCl_3$) at room temperature with tetramethylsilane (TMS) as an internal standard (Merck, Darmstadt, Germany). UV-Vis spectra were recorded on a Shimadzu UV-1800 spectrophotometer (Japan) at the 200-800 nm wavelength range. Fourier Transform infrared spectroscopy (FTIR) was done on a Perkin-Elmer Universal ATR Spectrometer (USA). Gas chromatography-mass spectrometry (GC-MS) was performed on a Shimadzu GCMS-QP 2010SE instrument (Japan) equipped with a DB-5SIL MS (30 m × 0.25 mm) fused silica capillary column (0.25 µm film thickness).

1.4 Elemental analysis

All glassware and polyethylene bottles were prewashed with 10% HNO_3 and repeatedly rinsed with doubly distilled water. Digestion of the plant samples followed the same procedure as described in our previous study [1]. Digestions were performed using the Microwave Accelerated

Reaction System (MARS 6, CEM Corporation, Matthews, NC, USA) with patented Xpress^{TM} Plus technology. Prior to digestion, 0.25 g crushed and dried samples were accurately weighed into 50 mL liners and 10 mL of 70% HNO_3 was added. The samples were allowed to predigest for 20 min. The liners were capped and carefully placed into the vessels. The vessels were then placed in the microwave system. The power was set at 100% at 1600 W; the temperature gradually increased to 180 °C for 15 min and remained at 180 °C for another 15 min. The vessels were then cooled for 15 min. Digested samples were transferred into 25 mL volumetric flasks diluted to the mark with doubly distilled water and stored in polyethylene bottles until elemental analysis. All determinations were done in quadruplicate. A sample blank containing 10 mL of HNO_3 was placed with each set of samples.

Analysis of elements (As, Ba, Ca, Cd, Co, Cr, Cu, Fe, Mg, Mn, Ni, P, Pb, Se and Zn) in the digested samples was obtained by inductively coupled plasma-optical emission spectrometry (ICP-OES) (Optima 5300DV, Perkin Elmer, Shelton, CN, USA) and reported as milligrams per kilogram based on dry mass. ICP standards solutions (1000 mg L^{-1} in 2% HNO_3) (Merck, Darmstadt, Germany) for calibration were of analytical grade. All working standards were made up with doubly distilled water and 10 mL of 70% HNO_3 to match the sample matrix.

Quality control was conducted by the use of both blank samples and the certified reference material (CRM) (White Clover, BCR 402) from the Community Bureau of Reference of the Commission of the European Communities. The samples were analyzed four times. The data was statistically evaluated using the statistical mean and standard deviation. The measured values were in good agreement with certified values which were (mean (SD) of measured value, certified value), for Fe (250 (18.0), 244 mg kg^{-1}), Se (6.81 (1.40), 6.70 (0.25) mg kg^{-1}) and Zn (30.3 (6.37), 25.2 mg kg^{-1}).

1.5 Antioxidant and antidiabetic properties

1.5.1 Chemicals and reagents

Alpha-glucosidase type I (EC 3.2.1.20, lyophilized powder) from *Saccharomyces cerevisiae*, α-amylase type VI-B (EC 3.2.1.1, lyophilized powder) from porcine pancreas, acarbose, ascorbic acid and α-tocopherol were purchased from Sigma Aldrich chemicals (St Louis, USA). *p*-Nitrophenyl-α-D-glucopyranoside (*p*NPG) and 2,2-diphenyl-l-picrylhydrazyl (DPPH) were obtained from Merck (Darmstadt, Germany). All other chemicals were of analytical grade.

1.5.2 Antioxidant activity

1.5.2.1 DPPH radical scavenging activity

The radical scavenging ability of *L. alat*ipes and *O. tenax* was established by the DPPH method as previously described with some modifications [8]. About 2850 µL of 0.1 mM methanolic DPPH solution was thoroughly mixed with 150 µL of extracts or compounds of various concentrations (10 – 500 µg mL^{-1}) then placed in the dark for 30 min at room temperature. The absorbance of the samples and blank was measured at 517 nm using a UV spectrophotometer (Biochrom Libra S11, Cambridge, England). Methanol served as the blank. Ascorbic acid and α-tocopherol were used as positive controls. The IC_{50} values (concentration of sample resulting in 50 % inhibition) were estimated from the dose-response curves. All experiments were done in triplicate. The percentage DPPH radical scavenging activity was calculated as follows

$$\% \text{ DPPH radical scavenging activity} = \left[\frac{A_c - A_S}{A_c}\right] \times 100 \qquad (3)$$

where A_C is the absorbance of the control (DPPH without the sample) and A_S is the absorbance of the sample.

1.5.2.2 Ferric reducing power

The reduction of Fe^{3+} to Fe^{2+} by *L. alatipes*, *O. tenax* and compounds was measured by the previously described FRAP method [9]. In brief, 1 mL of extract or compound of various concentrations (10 – 500 μg mL^{-1}) was mixed with 2.5 mL of 0.2 M phosphate buffer (pH 6.6) and 2.5 mL of 1% (w/v) potassium ferricyanide followed by incubation at 50 °C for 20 min. After incubation, 2.5 mL of 10% trichloroacetic acid (TCA) was added into the mixture followed by vortex mixing for 5 min. The mixture was centrifuged at 5000 rpm for 10 min. About 2.5 mL of the upper layer was mixed with 2.5 mL of doubly distilled water and 0.5 mL of 0.1% ferric chloride then left to stand for 10 min. The absorbance of the samples and blank was measured at 700 nm using a UV spectrophotometer (Biochrom Libra S11, Cambridge, England). A mixture of reagents and MeOH without the sample served as a blank. Ascorbic acid and α-tocopherol were used as positive controls. Experiments were conducted in triplicate.

1.5.3 Enzyme inhibitory activities

1.5.3.1 Alpha amylase

The α-amylase inhibition assay was established by a method previously described with some modifications [10]. Plant extracts or isolated compounds at various concentrations (200 μL) were added to sodium phosphate buffer (20 mM, pH 6.9, 250 μL) containing NaCl (0.006 M) and porcine pancreatic amylase (0.5 mg mL^{-1}, 250 μL) and incubated at 25 °C for 10 min. To the reaction mixture 500 μL of 1% starch solution (in 20 mM sodium phosphate buffer with 0.006 M NaCl, pH 6.9) was added and further incubated at 25 °C for 10 min. To terminate the reaction 1mL of 3,5-dinitrosalicylic acid (DNS) was added and the mixture was placed in a boiling water bath for 10 min then cooled to room temperature. The mixture was then diluted with 10 mL distilled water and the absorbance was measured at 540 nm using a UV spectrophotometer (Biochrom Libra S11, Cambridge, England). A solution containing the reagents and enzyme

without the sample was used as the control. Acarbose was used as a positive control. All experiments were conducted in triplicate. The IC_{50} values were estimated from the dose-response curves.

1.5.3.2 Alpha glucosidase

The α-glucosidase inhibition assay was established by a method previously described with some modifications [10]. Experiments were conducted in triplicate. Plant extracts or compounds at various concentrations (200 µL) were added to 100 µL of 0.5 mg mL^{-1} of α-glucosidase (in 0.1 M phosphate buffer, pH 6.9) and incubated at 25 °C for 10 min. Thereafter, 50 µL of 5 mM *p*-nitrophenyl-α-D-glucopyranoside (in 0.1 M phosphate buffer, pH 6.9) was added and incubated further at 25 °C for 5 min. The absorbance was measured at 405 nm using a UV spectrophotometer (Biochrom Libra S11, Cambridge, England). A solution containing the reagents and enzyme without the sample was used as the control. Acarbose was used as a positive control. The IC_{50} values were estimated from the dose-response curves.

The percentage inhibition was calculated as follows:

$$\% \text{ Inhibition} = \left[\frac{A_c - A_s}{A_c}\right] \text{x } 100 \qquad (4)$$

where A_C is the absorbance of the control and A_S is the absorbance of the sample.

1.6 Statistical analysis

Experimental data are expressed as mean (standard deviation). Statistical analyses were performed using the Statistical Package for the Social Sciences (PASW Statistics, Version 25, IBM Corporation, Cornell, New York). Significant differences between means were established by Tukey's Post-hoc tests ($p < 0.05$). Statistical analyses for antioxidant and antidiabetic activity were done using GraphPad Prism 5.0 (GraphPad Software Inc., San Diego, CA). One-way Anova was performed on the data followed by Dunnett's test ($p < 0.05$).

1.7 Nuclear magnetic resonance spectra (^{1}H-NMR and ^{13}C-NMR) of isolated compounds

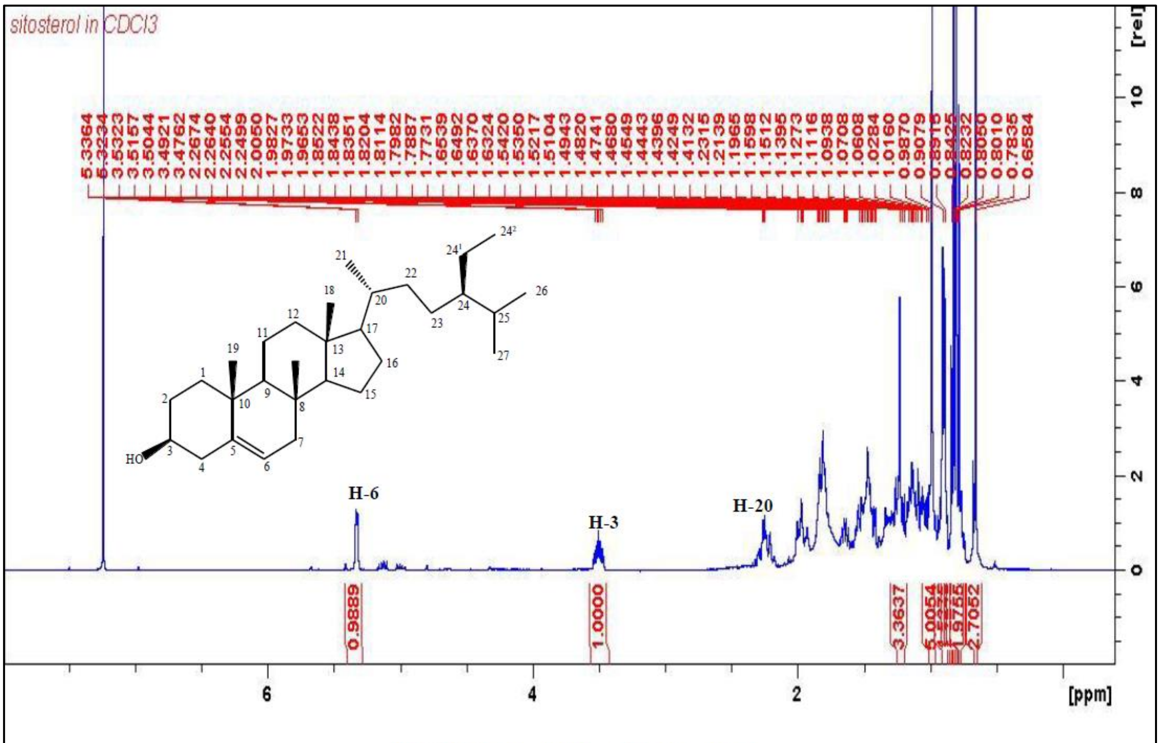

^{1}H-NMR spectrum of β-sitosterol

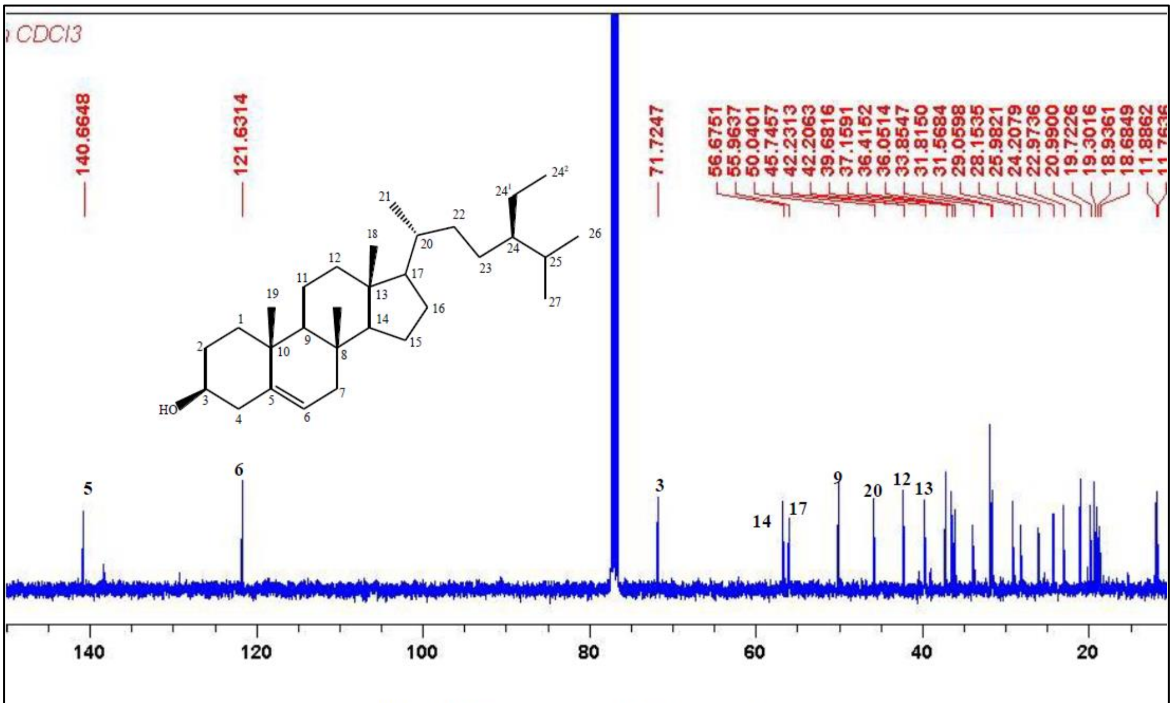

^{13}C-NMR spectrum of β-sitosterol

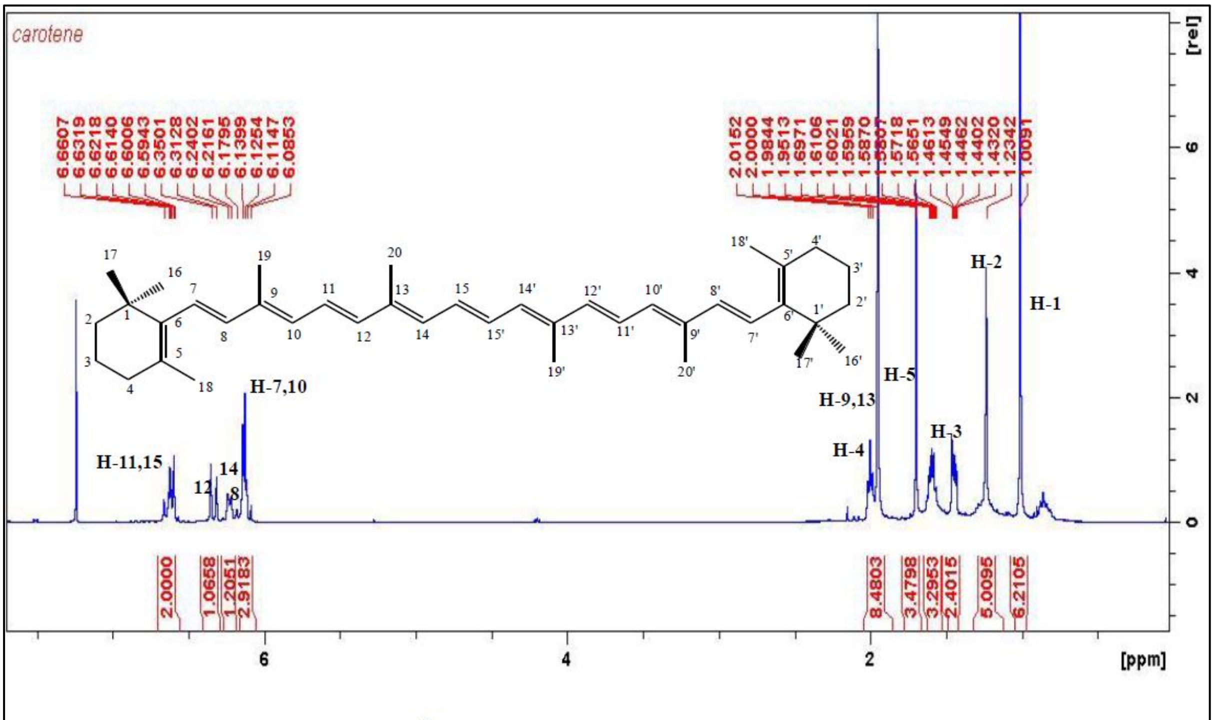

^{1}H-NMR spectrum of β-carotene

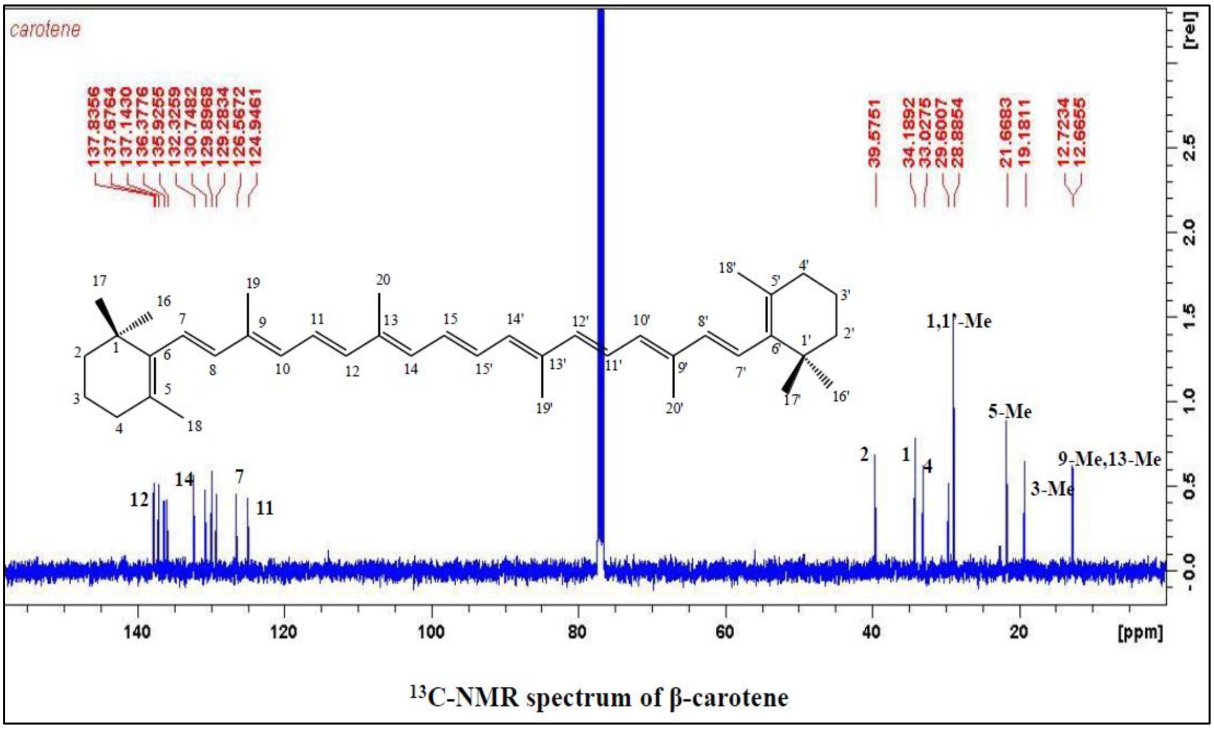

^{13}C-NMR spectrum of β-carotene

References

1. Mahlangeni, N. T., Moodley, R. & Jonnalagadda, S. B. The distribution of macronutrients, anti-nutrients and essential elements in nettles, *Laportea peduncularis* susp. *peduncularis* (River nettle) and *Urtica dioica* (Stinging nettle). *J. Environ. Sci. Health, Part B.* **51**, 160–169 (2016).
2. Elhassan, G. O. M. & Yagi, S. M. Nutritional composition of *Grewia* species fruits. *Adv. J. Food Sci. Technol*. **2**, 159–162 (2010).
3. Bradley Jr, R. L. Moisture and total solids analysis in *Food analysis* (ed. Nielsen, S. S.) 87–91 (Springer US, 2010).
4. Skoog, D. A., West, D. M., Holler, F. J. & Crouch, S. R. *Fundamentals of analytical chemistry*. (Thomson Brooks/Cole, 2004).
5. Igwemmar, N. C., Kolawole, S. A. & Imran, I. A. Effect of heating on vitamin C content of some selected vegetables. *Int. J. Sci. Technol. Res.* **2**, 209–212 (2013).
6. Besler, H. T., Çomoğlu, S. & OkÇu, Z. Serum levels of antioxidant vitamins and lipid peroxidation in Multiple Sclerosis. *Nutr. Neurosci.* **5**, 215–220 (2002).
7. Desai, I. D. Vitamin E analysis methods for animal tissues. *Methods Enzymol.* **105**, 138–147 (1984).
8. Saeed, N., Khan, M. R. & Shabbir, M. Antioxidant activity, total phenolic and total flavonoid contents of whole plant extracts *Torilis leptophylla* L. *BMC Complementary Altern. Med.* **12**, 1–12 (2012).
9. Sepahpour, S., Selamat, J., Manap, M. Y. A., Khatib, A. & Razis, A. F. A. Comparative analysis of chemical composition, antioxidant activity and quantitative characterization of some phenolic compounds in selected herbs and spices in different solvent extraction systems. *Molecules*. **23**, 1–17 (2018).

10. Shanmugam, S. & Thangaraj, P. *In vitro* antioxidant, antimicrobial and anti-diabetic properties of polyphenols of *Passiflora ligularis* Juss. fruit pulp. *Food Sci. Hum. Wellness*. **3**, 56–64 (2014).
